# Application of mathematical statistics to shale gas-bearing property evaluation and main controlling factor analysis

**DOI:** 10.1038/s41598-022-13863-1

**Published:** 2022-06-14

**Authors:** Min Li, Xiongqi Pang, Liang Xiong, Tao Hu, Di Chen, Zhen Zhao, Shasha Hui

**Affiliations:** 1 State Key Laboratory of Petroleum Resources and Prospecting, China University of Petroleum (Beijing), Beijing, 102249 China; 2 College of Geosciences, China University of Petroleum (Beijing), Beijing, 102249 China; 3 Research Institute of Exploration and Development, SINOPEC Southwest Branch Company, Chengdu, 610041 China

**Keywords:** Energy science and technology, Mathematics and computing

## Abstract

Gas-bearing property evaluation and main controlling factor analysis have remained a concern in shale gas research. The application of principal component analysis, an important mathematical statistical method, in gas-bearing property evaluation and main controlling factor analysis of the Longmaxi shale in the Weirong area, Sichuan Basin, was examined. The Longmaxi shale exhibits high heterogeneity, manifested in the organic matter abundance, mineral composition, and pore structure. Seven geological factors, including the temperature, pressure, TOC content, clay content, brittle mineral content, pore volume, and specific surface area (SSA), were selected in principal component analysis. Four principal components with geological significance, such as mineral composition, formation condition, pore structure, and organic matter abundance, were extracted through principal component analysis, and further constituted a comprehensive factor. Shale gas-bearing properties were evaluated according to the score of the comprehensive factor. The Longmaxi shale could be identified as exhibiting good, medium, and poor gas-bearing properties based on the comprehensive factor scores of these samples. According to each geological factor’s contribution to the comprehensive factor, combined with geological analysis, it could be considered that gas-bearing properties are primarily controlled by pore volume, SSA, and clay content, followed by TOC content, brittle mineral content, temperature and pressure.

## Introduction

Organic-rich shale is both a source rock and unconventional reservoir^1^. As an unconventional natural gas reservoir, shale can not only store free gas but can also adsorb gas^2,3^. Gas adsorption is an important property of shale reservoirs, and adsorbed and free gas fractions jointly largely account for the shale gas content and production^4,5^. Free gas is commonly distributed in large inorganic and organic pores and microcracks, whereas adsorbed gas generally occurs on the walls of organic pores and clay mineral pores with large specific surface areas^6^. In addition, heterogeneity is an inherent attribute of shale reservoirs, which is manifested in sedimentary facies and lithofacies at the macrolevel and mineral composition, organic geochemical characteristics, and pore structure at the microlevel^7^. Shale heterogeneity can lead to significant differences in gas-bearing properties.

The shale gas-bearing property is an essential index for shale gas exploration potential evaluation and sweet spot prediction^8,9^. The evaluation objects in regard to the shale gas-bearing property can be classified into three types, namely, total gas content evaluation, evaluation of the adsorbed gas or free gas content, and gas saturation evaluation^10,11^. The evaluation methods for the shale gas-bearing property can be classified into three types, including the experiment/field testing method^12–16^, geophysical method^17–19^, and mathematical statistical method^20,21^. For example, Chen et al.^18^ evaluated shale gas-bearing properties and predicted the spatial distribution based on well log interpretation and seismic multiple-attribute analysis. In addition, Tang et al.^20^ first combined principal component analysis and gray relational analysis to determine the main controlling factors of the free gas and adsorbed gas contents and then used multiple regression analysis to establish a quantitative relationship between the free gas content, adsorbed gas content and main controlling factors to realize shale gas-bearing evaluation. Similarly, Chen et al.^21^ applied isotopic geochemical proxies in shale gas content prediction. In another work, Li et al.^22^ used the methane isothermal adsorption experiment and field gas desorption methods to obtain the adsorbed gas content and total gas content, respectively. Similarly, Xiao et al.^23^ calculated the adsorbed gas content and total gas content according to the Langmuir adsorption model and gas state equation and recovered the total gas content based on the AMOCO method, indicating that the calculated gas content suitably agreed with the recovered gas content.

Effective evaluation of the shale gas-bearing property has remained a concern. Literature review revealed that various experimental tests or geophysical methods were the most commonly used to evaluate gas-bearing properties. The experimental test method provides the advantages of convenient operation and intuitive results, but due to the limitations of the corresponding experimental temperature and pressure conditions, the in situ gas content cannot be directly obtained^24^. The advantage of a geophysical method is that this approach can widely evaluate shale gas-bearing properties. However, the disadvantage of this method is that the accuracy of the evaluation results depends on the quality of logging or seismic data^25^. Compared to the first two methods, the mathematical statistical method is simpler, more straightforward and more widely applicable. Although the accuracy of the evaluation model depends on whether the number of statistical samples is sufficient and whether the geological factors are comprehensively considered, these problems can be overcome via the addition of samples and geological factors. Thus, this study examined the application of principal component analysis, a mathematical statistical method, in shale gas-bearing evaluation of the Longmaxi Formation in the Weirong area, southern Sichuan Basin. In contrast to other mathematical statistical methods, such as multiple regression, this method is more comprehensive and can simultaneously realize gas-bearing evaluation and main controlling factor analysis. Moreover, this study provides a feasible method for intelligent evaluation of shale gas-bearing properties.

## Geological setting

**Figure 1:**
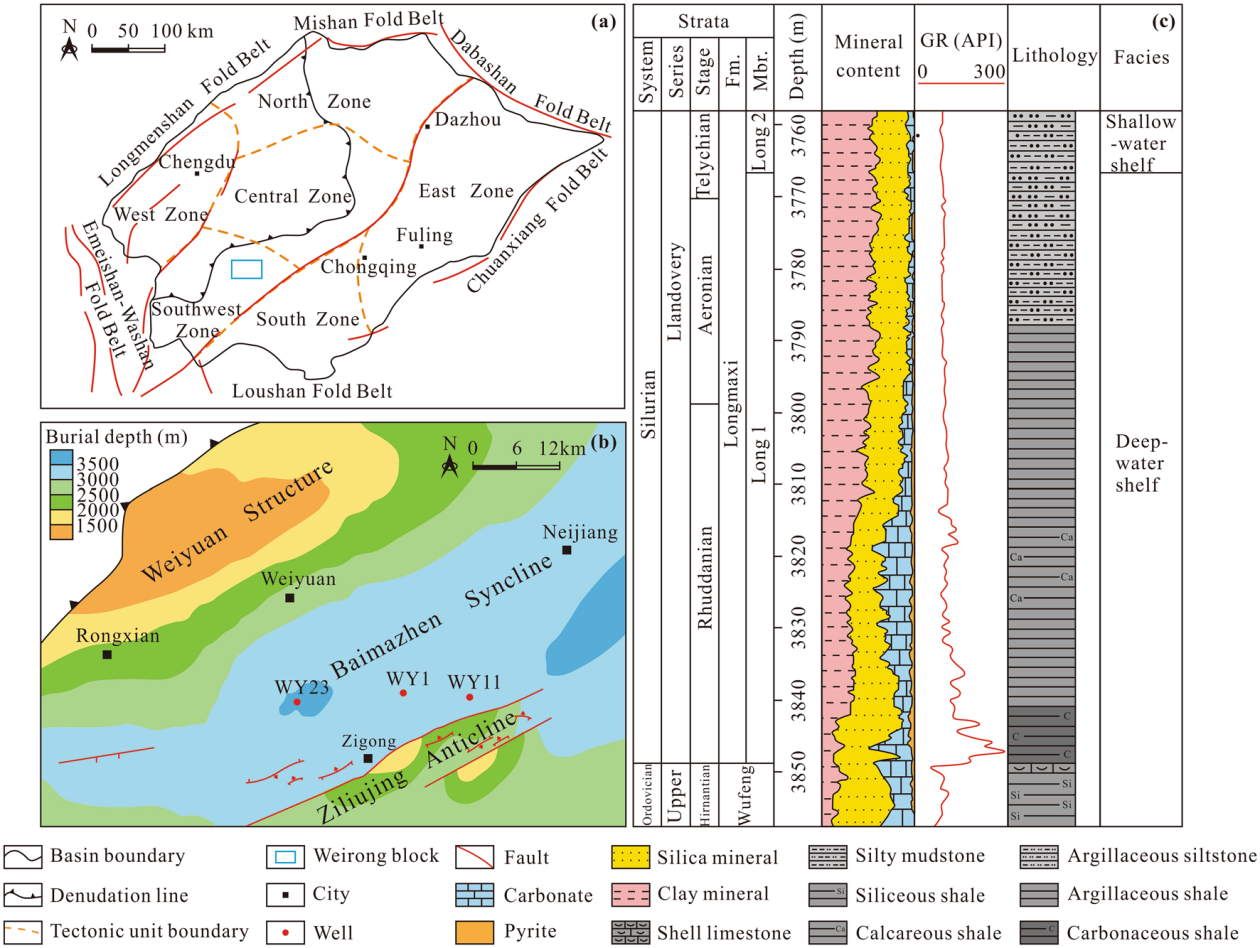
(**a**) Location of the Weirong area in the Sichuan Basin (modified after Nie et al.^29^); (**b**) Sampling well location in the Weirong block (modified after Xiong^30^); (**c**) Stratigraphic information of the Longmaxi Formation (modified after Xu et al.^31^).

The Weirong block is located in the southern Sichuan Basin (Fig. 1). The basement of the Sichuan Basin comprises Middle and Upper Proterozoic strata, on which Sinian to Jurassic strata were successively deposited except for the lack of Devonian and Carboniferous strata. The Longmaxi Formation contains a set of marine shales deposited during the early Silurian^26^. The Longmaxi Formation can be divided into two members based on the lithology and biological characteristics^27^. The first member of the Longmaxi Formation (S_1_l_1_) comprises gray black and black carbonaceous shale, with abundant graptolite and radiolarian fossils. The lithology of the second member (S_1_l_2_) primarily indicates dark gray and grayish-green shale and silty shale, and biological fossils are underdeveloped. Vertically, the lithological change in the Longmaxi Formation indicates a trend whereby the sand content gradually increases and the clastic grain size increases from the bottom up, reflecting the change in the sedimentary environment from deep to shallow-water shelf environments. The Longmaxi Formation was uplifted and denuded in a large area during the late Silurian. This formation is mainly distributed in the east, south, and southwest of the Sichuan Basin, with a residual area of approximately 14.4 × 10^4^ km^2^. The burial depth of the Longmaxi Formation in the Weirong area ranges from approximately 3000–3500 m. The thickness of the organic-rich shale of S_1_l_1_ ranges from 80 to 90 m, which is the focus of shale gas exploration. Exploration and drilling results suggest that the Longmaxi Formation in the Weirong block is generally subject to a high pressure, and the pore fluid pressure coefficient varies between ~ 1.94 and 2.05^28^, which provides suitable conditions for shale gas preservation but also causes great difficulties in fracturing development of shale gas.

## Materials and methods

### Source rock and reservoir parameters

The Longmaxi shale samples from well WY1, WY11, and WY23 were analyzed for experimental data in the Experimental Research Center of Wuxi Research Institute of Petroleum Geology, SINOPEC. TOC measurement, X-ray diffraction (XRD) analysis, porosity test, low-pressure nitrogen adsorption was carried out on sixty-one samples, respectively. The TOC content, mineral composition, porosity, pore structure parameters of these shale samples were obtained. Twelve samples were prepared for kerogen microscopic examination to analyze the organic matter type. Eight samples were tested for bitumen reflectance to obtain organic matter maturity. Forty-two samples were performed on a gas chromatograph to obtain the composition of natural gas. Three samples were measured for the carbon isotopic composition of methane. For the specific operation process of the above experiment, please refer to Han et al.^32^, Pei et al.^33^, and Wu et al.^34^.

### Total gas content measurement

The field desorption method directly measures the gas content of sixty-one shale samples. The core just taken out from the wellhead was quickly put into the desorption tank for shale gas natural desorption. When shale gas desorption, the mud circulation temperature is adopted for the first 3 h to simulate the gas desorption during coring. Then the temperature in the next 6–8 h is set to 110 °C. At this temperature, the residual gas is ignored. The desorbed gas volume was observed and recorded at different times. The gas desorption ends when the reading change of high-precision flowmeter is no more than 0.1 cm^3^. The lost gas volume was recovered by the USBM method. The measured desorption gas volume and calculated loss gas volume add up to the total gas content.

### Principal component analysis

Principal component analysis, a mathematical statistical method, involves the reduction in the dimensions of multiple variables^35^. The principle of this method entails the transformation of numerous possibly correlated variables into fewer linearly uncorrelated variables through orthogonal transformation^36^. The transformed variables are referred to as principal components, which can effectively reflect the information of the original variables. This study used principal component analysis to evaluate shale gas-bearing properties. The influencing factors of gas-bearing properties can be divided into three types: the first type includes gas generation conditions, such as the organic matter abundance and maturity; the second type includes gas storage conditions, such as the pore volume and specific surface area; and the third type includes gas preservation conditions, such as the formation pressure and fault development degree^37–40^. Combined with the actual geological features of the Longmaxi shale, seven original variables, including the temperature, pressure, TOC, clay content, brittle mineral content, pore volume, and specific surface area (SSA), were considered. The steps of principal component analysis are as follows:

1. Primitive variable *X*_b_ (b = 1, 2, …, 7) can be standardized as *Y*_b_ (b = 1, 2, …, 7) to reduce dimensions and eliminate order of magnitude differences among the various primitive variables, Eqs. (1)–(3).
  1 $$y_{{{\text{ab}}}} = \frac{{x_{ab} - \overline{x}_{b} }}{{s_{b} }},a = 1,2, \cdots ,61;\;\;b = 1,2, \ldots ,7$$
  2 $$\overline{x}_{b} = \frac{1}{61}\sum\limits_{a = 1}^{61} {x_{ab} } ,\;\;b = 1,2, \ldots ,7$$
  3 $$s_{b} = \sqrt {\frac{{\sum\nolimits_{a = 1}^{61} {(x_{ab} - \overline{x}_{b} )^{2} } }}{60}} ,\;\;b = 1,2, \ldots ,7$$
  where *x*_ab_ is the value of the a-th sample of the b-th variable, $\overline{x}_{{\text{b}}}$ is the mean value of 61 samples of the b-th variable, *s*_b_ is the standard deviation of the 61 samples of the b-th variable, and *y*_ab_ is the value of the a-th sample of the b-th variable after standardization.
2. The correlation coefficient matrix *R* = (*r*_ab_)_7×7_ of the standardized data matrix *Y* = (*y*_ab_)_61×10_ can be calculated based on Eq. (4).
  4 $$r_{ab} = \frac{1}{60}\sum\limits_{k = 1}^{61} {y_{ka} y_{kb} } ,a = 1,2, \cdots ,7;\;\;b = 1,2, \ldots ,7$$
  where *r*_ab_ is the correlation coefficient between the a-th and b-th variables, *r*_aa_ = 1, and *r*_ab_ = *r*_ba_.
3. The eigenvalue *λ*_b_ (b = 1, 2, …, 7; *λ*_1_ ≥ *λ*_2_ ≥ … ≥ *λ*_7_ ≥ 0) and standard orthogonal eigenvector *β*_b_ (b = 1, 2, …, 7) of the correlation coefficient matrix *R* = (*r*_ab_)_7×7_ can be obtained. The information contribution rate *δ*_b_ (b = 1, 2, …, 7) and cumulative contribution rate *α*_b_ (b = 1, 2, …, 7) of *λ*_b_ (b = 1, 2, …, 7) can be calculated according to Eqs. (5)–(6). For *α*_k_ ≥ 0.90, this indicates that the first k new variables can fully express the information of the original variables. Thus, k principal components *F*_b_ (b = 1, 2, …, k; k ≤ 7) can be extracted.
  5 $$\delta_{b} = \frac{{\lambda_{b} }}{{\sum\nolimits_{a = 1}^{7} {\lambda_{a} } }},\;\;b = 1,2, \ldots ,7$$
  6 $$\alpha_{k} = \frac{{\sum\nolimits_{b = 1}^{k} {\lambda_{b} } }}{{\sum\nolimits_{a = 1}^{7} {\lambda_{a} } }},1 \le k \le 7,k \in Z$$
4. Principal component *F*_b_ (b = 1, 2, …, k; k ≤ 7) is the product of eigenvector *β*_b_ (b = 1, 2, …, k) and standardized variable *Y*_b_ (b = 1, 2, …, 7) (Eq. (7)). The comprehensive score (*E*) can be calculated based on Eq. (8).
  7 $$F_{b} = \beta_{b1} Y_{1} + \beta_{b2} Y_{2} + \cdots + \beta_{b7} Y_{7} ,\;\;b = 1,2, \ldots ,k$$
  8 $$E = \sum\limits_{b = 1}^{k} {\delta_{b} } F_{b}$$

### Workflow

**Figure 2:**
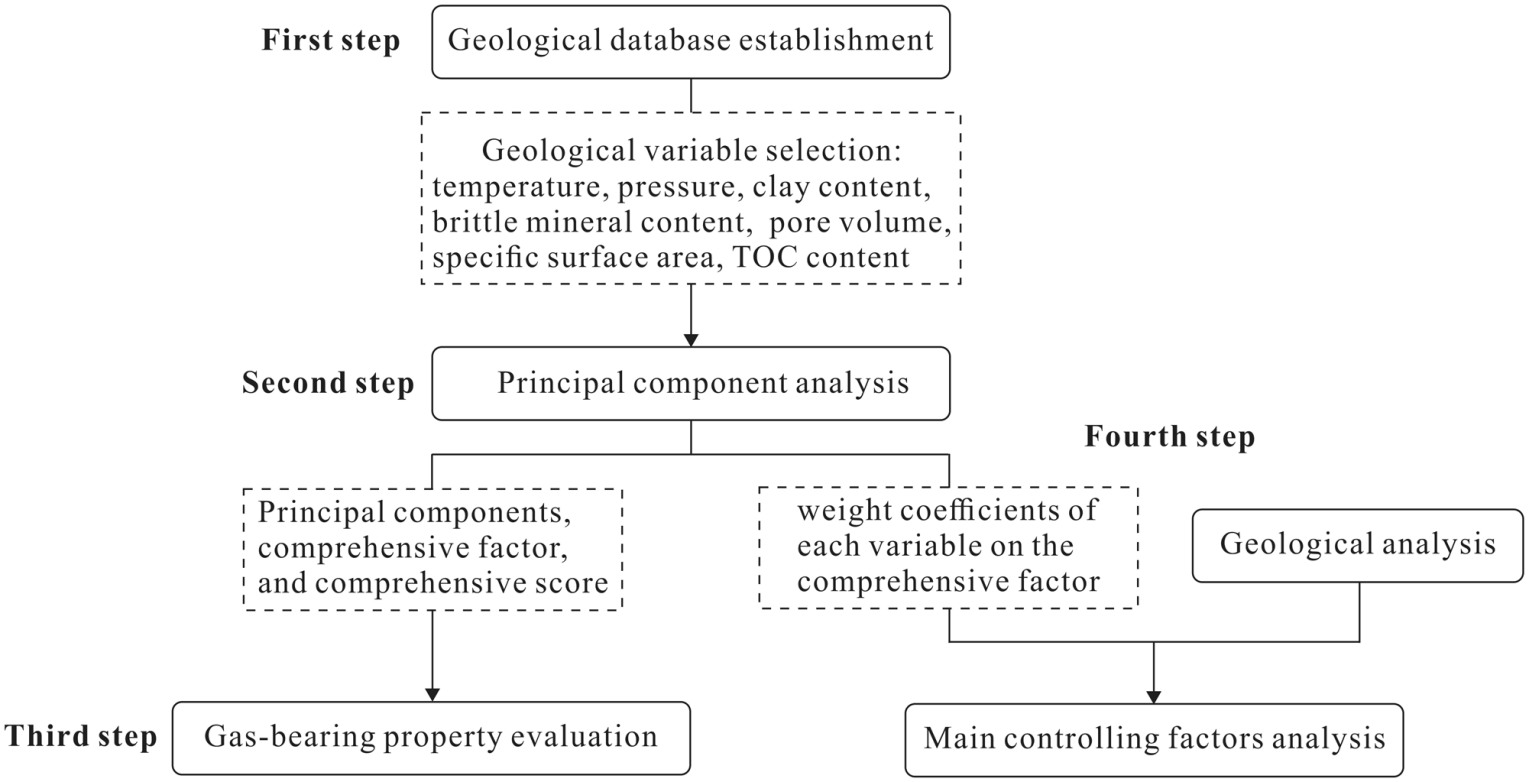
Flow chart of gas-bearing property evaluation and main controlling factor analysis.

This study was conducted in four steps (Fig. 2). The first step was the establishment of a geological database of the Longmaxi shale and selection of geological variables, including the formation temperature and pressure, clay content, brittle mineral content, pore volume, specific surface area, and TOC content. The second step entailed the substitution of values of these geological variables of the Longmaxi shale samples into SPSS software for principal component analysis. The principle and steps of principal component analysis are described in “Principal component analysis” section. Four principal components were extracted through principal component analysis, constituting a comprehensive factor. The comprehensive score of each shale sample and weight coefficient of each variable of the comprehensive factor could also be obtained. Based on the results of principal component analysis, third and fourth steps were performed. In the third step, the gas-bearing property could be evaluated based on the calculated comprehensive scores. Shale samples with high comprehensive scores exhibit good gas-bearing properties. Moreover, the gas content in shale could be quantitatively predicted by establishing a relationship between the comprehensive score and gas content. In the fourth step, the weight coefficient of each variable of the comprehensive factor could be determined, reflecting the relative impact of this factor on the gas-bearing property. Combining the weight coefficients of each variable with geological analysis, the main controlling factors of the gas-bearing property could be further determined.

## Results and discussion

### Establishment of a geological database

#### Organic geochemical characteristics

**Figure 3:**
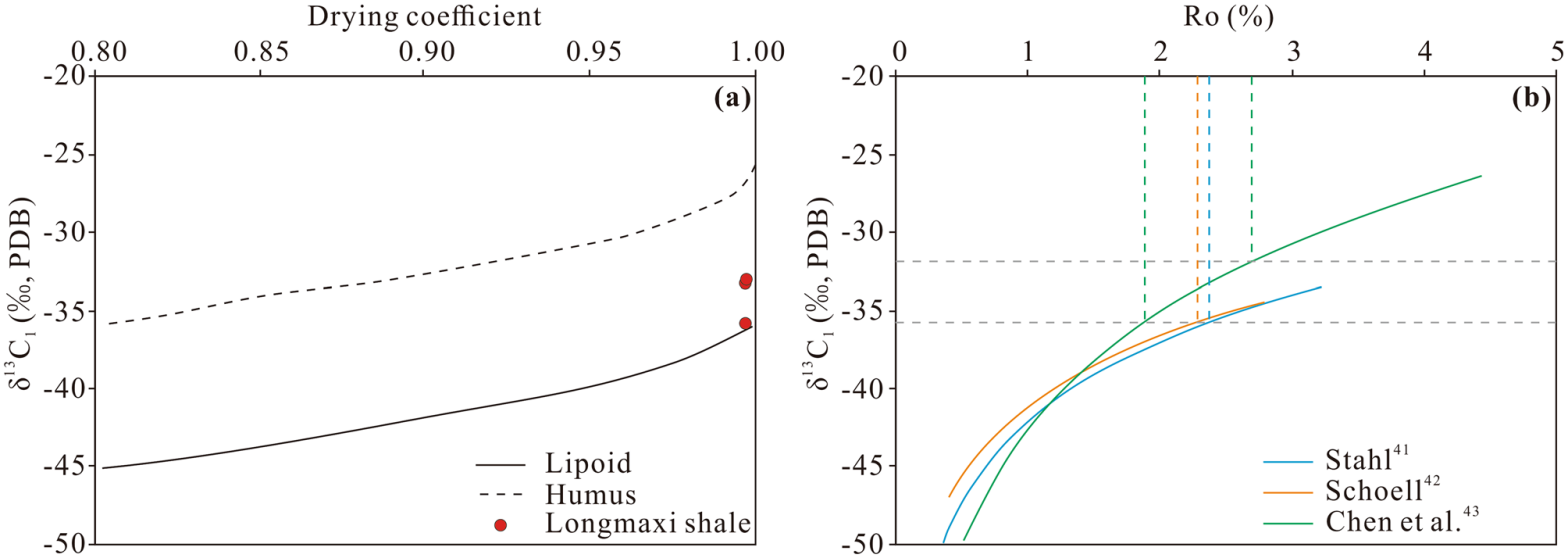
(**a**) Relationship between the carbon isotope composition of methane and hydrocarbon composition of natural gas generated by lipoids (modified after Stahl and Carey^46^ and Li et al.^47^). (**b**) Relationship between δ^13^C_1_‰ and Ro for oil-type gas (modified after Chen et al.^43^). The carbon isotopic composition of methane is mainly distributed between the ordinates corresponding to the two horizontal dotted lines. The maturity of the Longmaxi shale is distributed within the range between the abscissa corresponding to the two vertical dotted lines.

The kerogen macerals of the Longmaxi shale samples mainly include sapropelic amorphous and planktonic alginite but hardly include vitrinite. The type index (TI) value ranges from 78.22 to 100, indicating that the organic matter in the Longmaxi shale is dominated by type I kerogen (Table 1). The bitumen reflectance (R_b_) of the Longmaxi shale ranges from 2.72 to 3.22%, and the equivalent vitrinite reflectance (R_o_) ranges from 2.08 to 2.39%, demonstrating that the Longmaxi shale has generally entered the stage of overmature thermal evolution (Table 1). The results of gas chromatographic analysis reveal that methane is the main component of shale gas, with a methane content ranging from 73.82 to 97.72% and a drying coefficient higher than 95%. The stable carbon isotope composition of methane (δ^13^C_1_‰) ranged from approximately − 31.9‰ to − 35.8‰. According to the δ^13^C_1_‰ and shale gas composition results (Fig. 3a), it could be determined that the organic parent material is lipoid, which is consistent with the results of kerogen microscopic examination. Moreover, δ^13^C_1_‰ can provide information on the maturity of organic matter. Stahl^41^, Schoell^42^, and Chen et al.^43^ established quantitative relationships between δ^13^C_1_‰ and R_o_ for oil-type gas. Based on these relationships, the Longmaxi shale can be classified as generally overmature, with R_o_ exceeding 2.0% (Fig. 3b). The Longmaxi shale exhibits a TOC content ranging from 0.06 to 6.04 wt% (averaging 2.06 wt%), indicating a suitable shale hydrocarbon generation potential (Supplementary Table 1).

**Table 1:** Kerogen macerals and reflectance of the Longmaxi shale, southern Sichuan Basin.

Samples	Depth (m)	Sapropelite (%)		Exinite (%)	Vitrinite (%)	Inertinite (%)	TI	Kerogen type	R_b_ (%)	R_o_ (%)
		Amorphous	Alginite							
WY1-1	3526.95	99.31	0.23	–	0.46	–	99.86	I		
WY1-2	3541.02	97.70	0.46	0.23	1.61	–	99.08	I		
WY1-3	3556.73	98.85	0.23	–	0.92	–	99.54	I		
WY1-4	3568.54	75.25	0.49	–	24.26	–	87.87	I		
WY1-5	3587.21	56.43	–	–	43.57	–	78.22	II_1_	3.22	2.39
WY11-1	3688.80	65	35	–	–	–	100	I	2.88	2.18
WY11-6	3719.18	45	55	–	–	–	100	I	2.72	2.08
WY11-24	3763.52	95	5	–	–	–	100	I	2.84	2.16
WY23-7	3805.94	85	15	–	–	–	100	I	2.75	2.10
WY23-20	3828.82	95	5	–	–	–	100	I	2.83	2.15
WY23-29	3842.93	94	6	–	–	–	100	I	2.89	2.19
WY23-34	3850.84	68	32	–	–	–	100	I	2.93	2.21

R_o_ is calculated with the following equation: *R*_o_ = 0.618*R*_b_ + 0.4^44^. The type index can be calculated with the following equation *TI* = 100 × *a* + 100 × *b*_1_ + 50 × *b*_2_ + 10 × *c*_1_ − 75 × *c*_2_ − 100 × *d*^45^, where *a* is the percentage of sapropelite, *b*_1_ is the percentage of resinite in exinite, *b*_2_ is the percentage of other exinite components, *c*_1_ is the percentage of perhydrous vitrinite, *c*_2_ is the percentage of normal vitrinite, and *d* is the percentage of inertinite. A blank column indicates that no data are measured. A dash indicates that the data value is zero.

#### Mineral compositions

**Figure 4:**
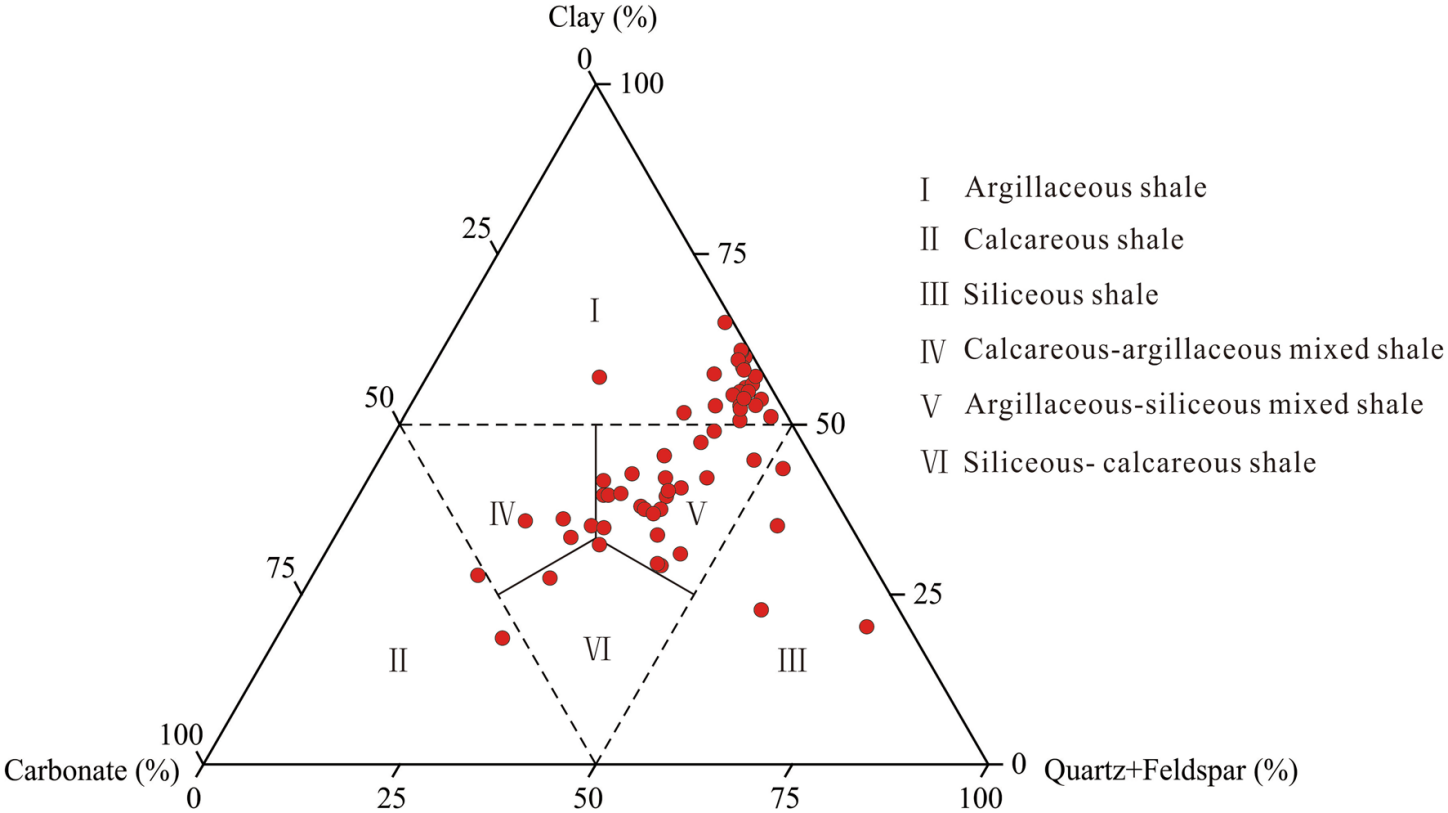
Lithofacies classification of the Longmaxi shale in the Weirong area (modified after Yuan et al.^48^).

The XRD analysis shows that the Longmaxi shale mainly contains clay minerals and quartz, and their average contents are 42.3% and 33.9%. Dolomite, calcite, pyrite, and feldspar are not abundant, with average contents of 8.4%, 7.7%, 3.5%, and 3.3%, respectively. Clay minerals are dominated by illite/ smectite mixed layer and illite. Illite/smectite mixed-layer accounts for 32.0–60.0% of clay minerals, with an average of 48.7%, whereas illite make up 33.0–50.0%, with an average of 41.6%. Chlorite and kaolinite account for only a small proportion of clay minerals. The Longmaxi shale primarily contains two lithofacies, namely argillaceous shale and argillaceous-siliceous mixed shale (Fig. 4). The argillaceous shale has more clay and silica minerals and less carbonate, with their contents in the range of 48.0–61.0% (average of 52.9%), 21.0–44.0% (average of 37.7%), and 1.0–20.0% (average of 4.1%), respectively. By contrast, the argillaceous-siliceous mixed shale contains less clay and silica minerals and more carbonate minerals, with their contents in the range of 28.0–47.0% (averaging 37.3%), 29.0–45.0% (averaging 36.6%) and 7.0–30.0% (averaging 21.3%).

#### Pore structure characteristics

**Figure 5:**
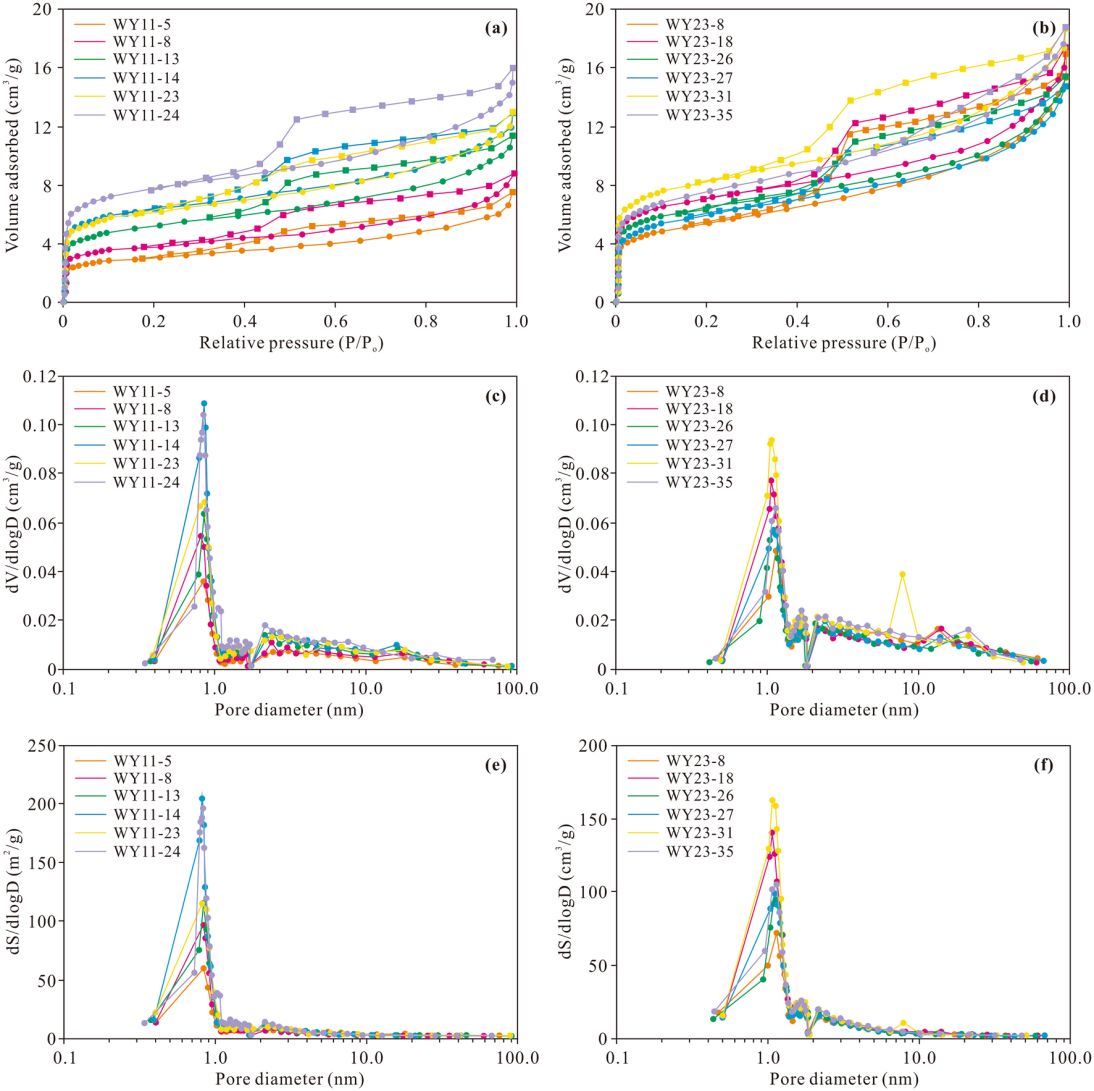
Nitrogen adsorption and desorption isotherm (**a**,**b**), pore volume change rate with the pore size (**c**,**d**), and SSA change rate with the pore size (**e**,**f**) of the Longmaxi shale samples in the Weirong area.

Nitrogen adsorption/desorption isotherms for twelve shale samples retrieved from two wells are shown in Fig. 5. These isotherms are characterized by an obvious hysteresis loop and Point B. The adsorption isotherm is convex under a low relative pressure, caused by monolayer coverage. When the adsorption isotherm is almost linear with the relative pressure, the single-layer adsorption process ends, and multilayer adsorption begins. The adsorption isotherm reveals a slightly concave trend under a high relative pressure and does not reach the limiting uptake. Then, the relative pressure drops, and the desorption isotherm does not coincide with the adsorption isotherm, forming a hysteresis loop associated with capillary condensation in pores. Based on the IUPAC classification^49^, the nitrogen adsorption/desorption isotherms belong to type IV isotherms, and the hysteresis loops are mainly of the H2 and H3 types. The information reflected by the isotherm shape indicates that the Longmaxi shale contains abundant mesopores, mainly ink bottle-shaped and slit-like pores.

**Figure 6:**
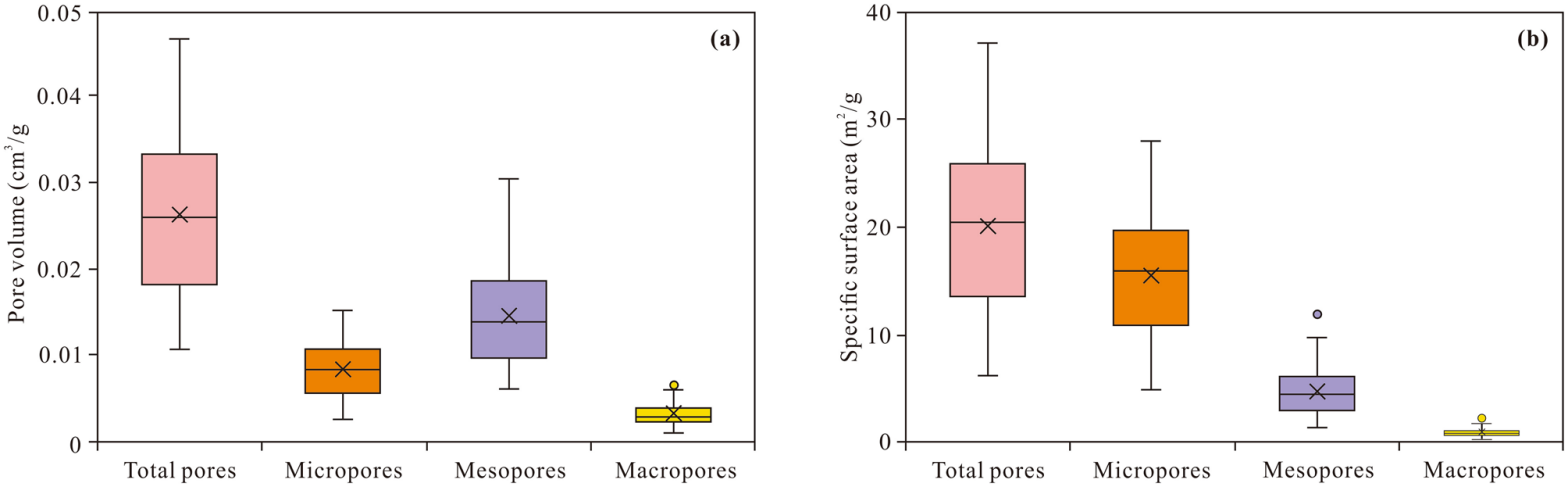
Pore volume (**a**) and SSA (**b**) of the micropores, mesopores, macropores and total pores of the Longmaxi shale in the Weirong area.

The change rate of the pore volume and SSA of the Longmaxi shale with the pore diameter is shown in Fig. 6. The pore volume obviously changes when the pore diameter ranges from 0.6 to 1.0 nm and 2.0 to 10.0 nm. The SSA obviously varies within the 0.6–1.0 nm pore size range. Thus, the pore size of the Longmaxi shale primarily ranges from 0.6 to 1.0 nm and 2.0 to 10.0 nm. In addition, a quantitative description of the pore structure can be obtained from the generated nitrogen adsorption/desorption isotherms. The pore volume and SSA of the shale range from 0.011 to 0.046 cm^3^/g (averaging 0.026 cm^3^/g) and 6.304 to 37.011 m^2^/g (averaging 20.009 m^2^/g), respectively (Supplementary Table 1). The pore volume of mesopores is the largest, with a mean value of 0.015 cm^3^/g, followed by micropores and macropores, with average values of 0.008 cm^3^/g and 0.003 cm^3^/g, respectively. Micropores exhibit the largest SSA, with an average value of 15.385 m^2^/g. In contrast, mesopores and macropores exhibit a smaller specific area. The average SSA values of mesopores and macropores are 4.585 m^2^/g and 0.039 m^2^/g, respectively.

### Application of principal component analysis

**Figure 7:**
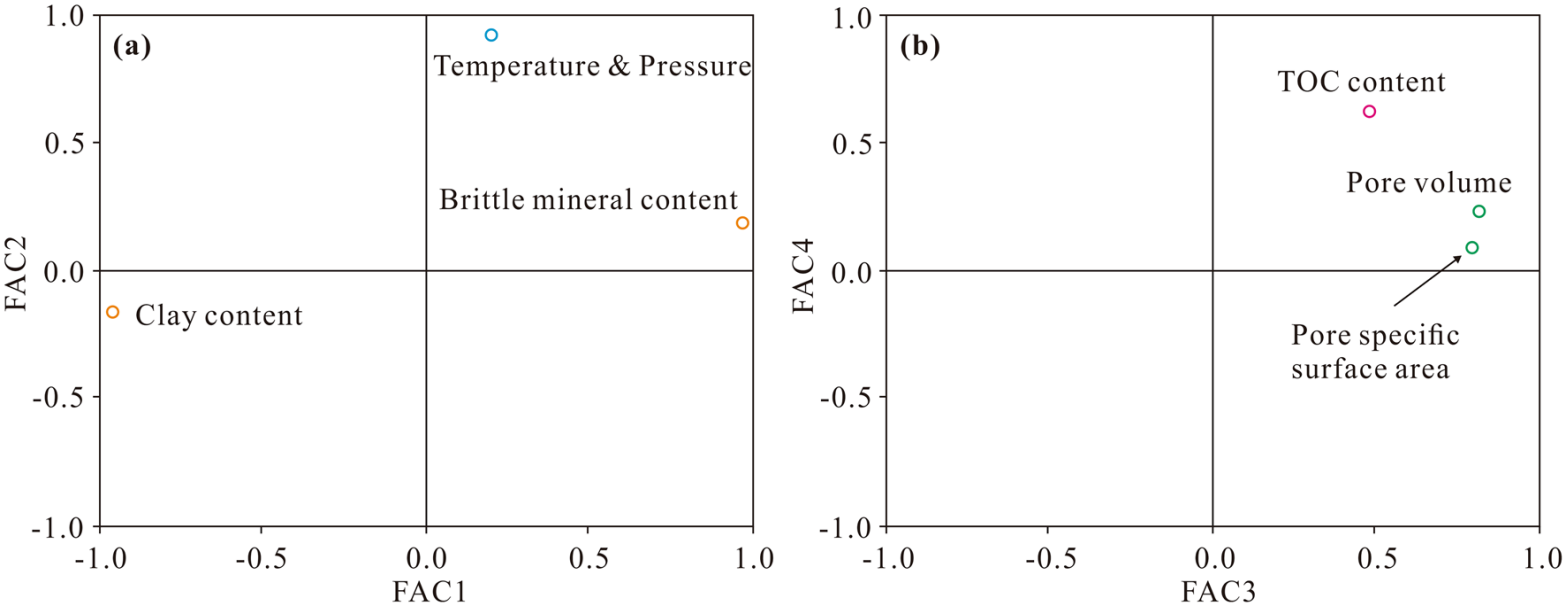
Principal components and related variables. (**a**) Variables related to FAC1 and FAC2; (**b**) variables related to FAC3 and FAC4.

Seven factors, including the TOC content, temperature, pressure, pore volume, specific surface area, clay content, and brittle mineral content, were selected from the geological database for principal component analysis (Supplementary Table 1). These factors are related to gas-bearing properties, and these data are easy to obtain. The results of principal component analysis are reliable since the Kaiser–Meyer–Olkin (KMO) value is 0.73, which is greater than 0.6, and the significance of Bartlett’s test of sphericity is lower than 0.05. Four principal components were extracted, namely, FAC1, FAC2, FAC3, and FAC4. Each principal component is a linear combination of the original variables. The weight coefficients of each variable of these four principal components are listed in Table 2. FAC1, FAC2, FAC3, and FAC4 captured 33.9%, 32.8%, 25.6%, and 7.1%, respectively, of the total variability. FAC1, FAC2, FAC3, and FAC4 could explain 99.4% of the total variability. Thus, we used these four principal components to evaluate the gas-bearing property. FAC1 exhibited a positive relationship with the brittle mineral content but was negatively correlated with the clay content (Fig. 7a). FAC2 was positively correlated with the temperature and pressure (Fig. 7a). FAC3 attained a positive relationship with the pore volume and specific surface area (Fig. 7b). FAC4 was positively correlated with the TOC content (Fig. 7b). Based on these observations, FAC1–FAC4 could reflect geological significance well and could be interpreted as the mineral composition, formation condition, pore structure, and organic matter abundance, respectively. A comprehensive factor (CFAC) was obtained based on FAC1, FAC2, FAC3, and FAC4. The scores of FAC1, FAC2, FAC3, FAC4, and CFAC of each sample were calculated according to Eqs. (9)–(13), the eigenvectors of all standardized variables in Eqs. (9)–(13) are listed in Table 2, and the results are provided in Table 3. The weight coefficients of each variable of CFAC are listed in Table 2. The first three factors contributing the most to CFAC were the pore volume, SSA, and clay content (Table 2).

9 $$FAC1_{sample} = - 0.080X_{1} - 0.080X_{2} - 0.184X_{3} - 0.036X_{4} - 0.016X_{5} - 0.563X_{6} + 0.629X_{7}$$

10 $$FAC2_{sample} = 0.695X_{1} + 0.694X_{2} - 0.014X_{3} - 0.195X_{4} - 0.329X_{5} + 0.067X_{6} - 0.083X_{7}$$

11 $$FAC3_{sample} = - 0.457X_{1} - 0.455X_{2} - 0.329X_{3} + 0.921X_{4} + 0.882X_{5} + 0.049X_{6} + 0.052X_{7}$$

12 $$FAC4_{sample} = 0.236X_{1} + 0.232X_{2} + 1.962X_{3} - 0.789X_{4} - 0.365X_{5} + 0.367X_{6} - 0.711X_{7}$$

13 $$CFAC_{sample} = 0.341FAC1 + 0.330FAC2 + 0.258FAC3 + 0.071FAC4$$

where X_1_–X_7_ are standardized variables; X_1_ denotes the formation temperature, K; X_2_ denotes the formation pressure, MPa; X_3_ denotes TOC, wt%; X_4_ denotes the pore volume, cm^3^/g; X_5_ denotes the pore surface area, m^2^/g; X_6_ denotes the clay content, %; and X_7_ denotes the brittle mineral content, %.

**Table 2:** Factor score coefficient matrix and variance explained by each factor.

Geological variables	FAC1	FAC2	FAC3	FAC4	Weight coefficient
Temperature	− 0.080	0.695	− 0.457	0.236	0.149
Pressure	− 0.080	0.694	− 0.455	0.232	0.149
TOC	− 0.184	− 0.014	− 0.329	1.962	0.131
Pore volume	− 0.036	− 0.195	0.921	− 0.789	0.167
Specific surface area	− 0.016	− 0.329	0.882	− 0.365	0.164
Clay content	− 0.563	− 0.067	0.049	0.367	0.167
Brittle mineral content	0.629	− 0.083	0.052	− 0.711	0.071
Variance explained	33.9%	32.8%	25.6%	7.1%	
Ratio of variance contribution^a^	34.1%	33.0%	25.8%	7.1%	

^a^The ratio of the variance contribution refers to the variance explained by a single principal component (FAC1, FAC2, FAC3 or FAC4) to the total variance explained by the first four principal components.

**Table 3:** Scores of the four principal components and CFAC of the Longmaxi shale samples.

Samples	FAC1	FAC2	FAC3	FAC4	CFAC	Samples	FAC1	FAC2	FAC3	FAC4	CFAC
WY11-1	− 0.761	− 1.856	− 0.536	− 0.601	− 1.053	WY23-6	− 0.858	0.576	− 0.060	− 0.562	− 0.156
WY11-2	− 1.061	− 1.553	− 0.555	− 0.123	− 1.026	WY23-7	− 1.051	0.604	0.173	− 0.062	− 0.118
WY11-3	− 1.072	− 1.218	− 1.061	− 0.239	− 1.059	WY23-8	− 0.660	0.673	0.138	− 0.657	− 0.013
WY11-4	− 0.974	− 1.201	− 0.957	− 0.032	− 0.978	WY23-9	− 1.300	0.795	0.099	− 0.109	− 0.162
WY11-5	− 0.792	− 1.250	− 0.674	0.113	− 0.849	WY23-10	− 1.180	0.718	0.466	− 0.375	− 0.069
WY11-6	− 0.385	− 1.308	− 0.412	− 0.360	− 0.695	WY23-11	− 0.492	0.644	0.441	− 0.761	0.107
WY11-7	− 0.324	− 1.209	− 0.591	− 0.446	− 0.694	WY23-12	− 1.191	0.417	1.268	0.380	0.089
WY11-8	− 0.672	− 1.177	− 0.450	0.328	− 0.711	WY23-13	0.119	0.597	0.108	0.258	0.284
WY11-9	− 0.395	− 0.846	− 1.158	0.003	− 0.714	WY23-14	0.726	0.257	1.225	− 1.406	0.552
WY11-10	− 0.970	− 0.822	− 0.813	0.369	− 0.786	WY23-15	1.011	− 0.220	3.110	− 3.594	0.828
WY11-11	− 0.607	− 0.939	− 0.390	0.122	− 0.609	WY23-16	− 1.422	0.247	2.635	− 1.165	0.202
WY11-12	− 0.519	− 0.943	− 0.316	0.123	− 0.561	WY23-17	0.346	0.502	0.915	− 0.947	0.455
WY11-13	1.186	− 1.246	0.076	− 0.674	− 0.036	WY23-18	0.434	0.754	0.033	0.252	0.422
WY11-14	0.829	− 1.323	0.525	− 0.106	− 0.026	WY23-19	0.816	0.599	0.635	− 1.022	0.569
WY11-15	1.844	− 1.260	0.017	− 0.486	0.181	WY23-20	− 0.855	0.071	2.624	1.454	0.517
WY11-16	1.218	− 1.076	0.191	− 0.568	0.069	WY23-21	− 0.040	0.464	1.388	0.901	0.563
WY11-17	0.959	− 0.803	− 0.405	− 0.377	− 0.071	WY23-22	0.758	1.116	− 0.359	− 0.887	0.470
WY11-18	0.344	− 0.897	0.213	0.106	− 0.116	WY23-23	− 1.174	0.835	0.934	2.083	0.265
WY11-19	0.513	− 0.886	− 0.030	0.650	− 0.080	WY23-24	− 0.369	1.045	0.269	1.022	0.361
WY11-20	0.140	− 0.710	0.147	− 0.744	− 0.200	WY23-25	− 0.738	1.651	− 1.044	0.584	0.063
WY11-21	0.637	− 0.449	− 1.072	0.238	− 0.194	WY23-26	0.048	1.393	− 0.632	0.241	0.328
WY11-22	0.630	− 0.506	− 0.768	− 0.017	− 0.153	WY23-27	0.070	1.507	− 0.790	− 0.302	0.295
WY11-23	0.039	− 0.770	0.088	1.382	− 0.121	WY23-28	0.294	1.383	− 0.598	0.106	0.408
WY11-24	1.367	− 1.239	0.783	1.269	0.348	WY23-29	0.783	1.290	− 0.447	− 0.397	0.548
WY11-25	1.377	− 0.916	− 0.368	2.244	0.227	WY23-30	1.661	0.541	0.641	2.485	1.084
WY11-26	1.494	− 0.841	0.094	0.069	0.260	WY23-31	0.633	1.055	− 0.071	2.224	0.701
WY23-1	− 0.576	0.477	− 1.303	− 1.030	− 0.450	WY23-32	1.590	0.533	1.018	1.653	1.097
WY23-2	− 1.239	0.716	− 1.513	− 0.373	− 0.604	WY23-33	2.710	2.037	− 2.909	− 1.732	0.716
WY23-3	− 1.829	0.683	− 0.834	0.081	− 0.608	WY23-34	1.006	1.106	0.534	− 0.223	0.830
WY23-4	− 1.289	0.702	− 0.818	− 0.467	− 0.452	WY23-35	0.368	1.371	− 0.005	0.317	0.599
WY23-5	− 1.155	0.106	1.152	− 0.213	− 0.073						

### Gas-bearing property evaluation

**Figure 8:**
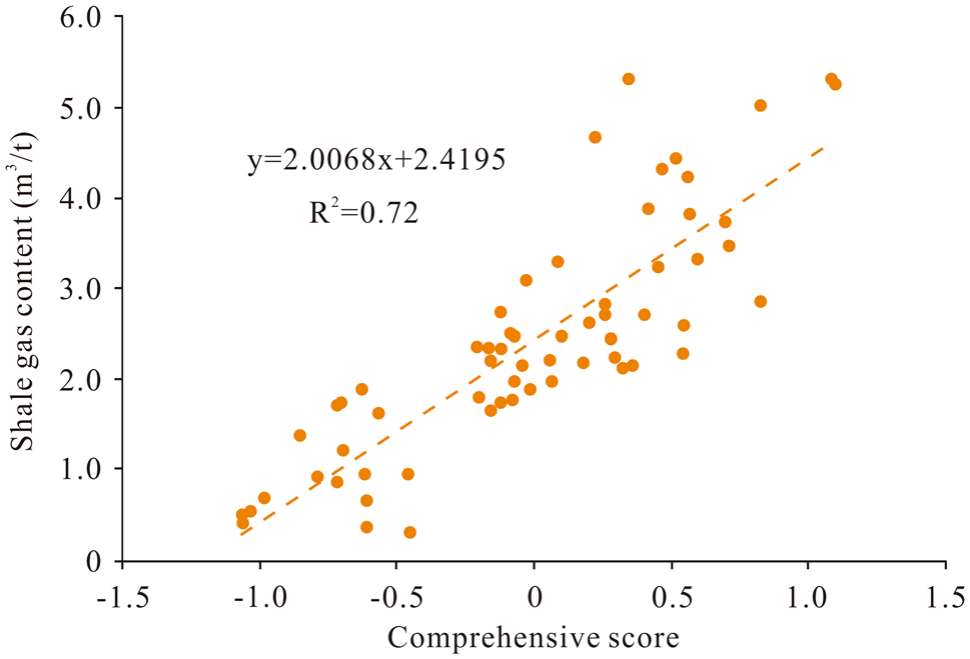
Scatter diagram of the positive relationship between the comprehensive score and gas content in the Longmaxi shale samples.

**Figure 9:**
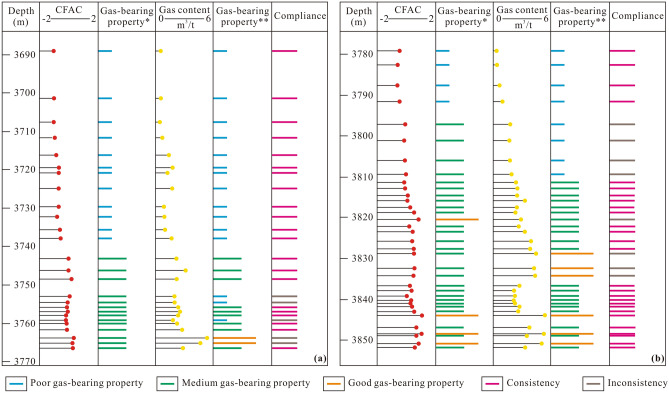
Verification of the gas-bearing property evaluation results based on the comprehensive score of the Longmaxi shale samples by the measured gas content. (**a**) Gas-bearing property evaluation results for well WY11; (**b**) gas-bearing property evaluation results for well WY23. *Indicates that the gas-bearing property is evaluated by the comprehensive score. **Indicates that the gas-bearing property is evaluated based on the measured gas content.

The measured gas content in the Longmaxi shale samples ranges from 0.31 to 5.32 m^3^/t. In general, shale with a gas content < 2 m^3^/t can be regarded as exhibiting poor gas-bearing properties, shale with a gas content ranging from 2 to 4 m^3^/t exhibits medium gas-bearing properties, and shale exhibits high gas-bearing properties given a gas content > 4 m^3^/t. In this study, the measured gas content in the Longmaxi shale samples was used to verify the principal component analysis results. The findings suggest that the comprehensive score obtained via principal component analysis is positively correlated with the measured gas content (Fig. 8). Therefore, the comprehensive score of principal component analysis can also be used to effectively evaluate the Longmaxi shale gas-bearing properties. A linear relationship between the comprehensive score and shale gas content was further established. The comprehensive scores corresponding to gas contents of 2 m^3^/t and 4 m^3^/t are − 0.209 and 0.788, respectively. Thus, shale with a score < − 0.209 can be evaluated as exhibiting poor gas-bearing properties, shale with a score ranging from − 0.209 to 0.788 exhibits medium gas-bearing properties, and shale with a score > 0.788 exhibits good gas-bearing properties. Gas-bearing property evaluation of a single well reveals that the results based on principal component analysis suitably agree with those based on the measured gas content, with the coincidence rates for wells WY11 and WY23 reaching 80.8% and 77.1%, respectively (Fig. 9). In addition, apparent differences in gas-bearing properties between these two wells are observed. Well WY11 exhibits poor gas-bearing properties, whereas the gas-bearing properties of well WY23 are primarily medium to good. This phenomenon may occur because the geological factors influencing the gas-bearing properties in these two wells are quite different.

### Main controlling factors of the gas-bearing properties

**Figure 10:**
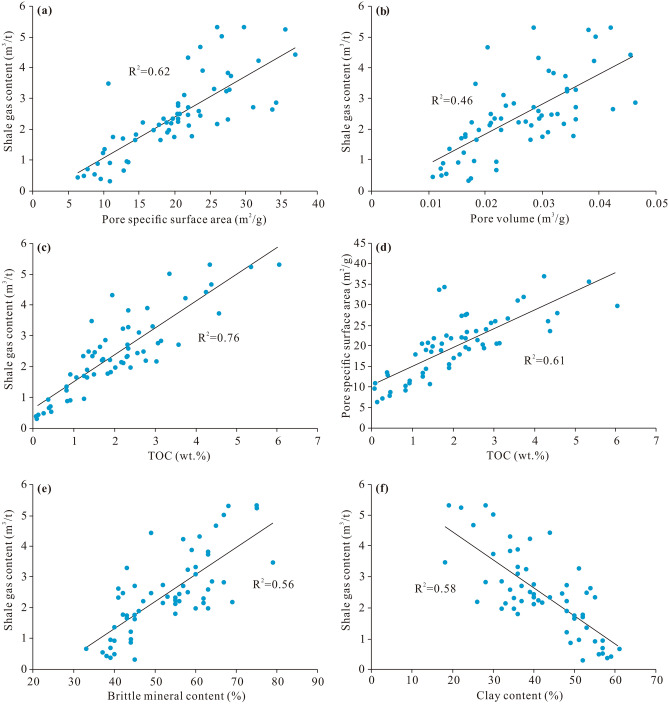
Influencing factors of shale gas-bearing properties. The correlations between SSA and shale gas content (**a**), between pore volume and shale gas content (**b**), between TOC content and shale gas content (**c**), between TOC content and SSA (**d**), between brittle mineral content and shale gas content (**e**), and between clay content and shale gas content (**f**), respectively.

The main controlling factors affecting the shale gas-bearing properties were revealed via principal component analysis. The above indicates that the geological factors contributing to the comprehensive factor include the pore volume, SSA, clay content, temperature, pressure, TOC content, and brittle mineral content, with the pore volume, SSA, and clay content yielding the greatest contribution (Table 2). Except for the clay content, which negatively contributes to the comprehensive factor, the other geological factors positively contribute to the comprehensive factor. Moreover, geological analysis reveals that most of these geological factors attain good relationships with the measured gas content, especially SSA, pore volume, TOC, brittle mineral content, and clay content (Fig. 10). The positive correlations among SSA, pore volume and gas content reflect the control of the pore structure on the gas-bearing properties (Fig. 10a,b), which is also shown in previous literature^50^. The pore volume and SSA primarily provide the occurrence space of free and adsorbed gas, respectively^51^. The larger the pore volume and SSA are, the more conducive these conditions to shale gas enrichment, which often indicates good gas-bearing properties^52^. Consistent with previous studies^53,54^, the TOC content exerts a positive effect on the gas content in this study (Fig. 10c), as reflected by the gas supply and storage. On the one hand, a high TOC content reflects a high gas generation potential, which is the premise of good gas-bearing properties. On the other hand, the TOC content is positively related to SSA (Fig. 10d), indicating that the organic pores developed within the organic matter provide a very large SSA for adsorbed gas. In addition, it has been revealed in previous studies that organic pores notably contribute to the pore volume in regard to free gas^55,56^. A change in mineral composition can also lead to a difference in shale gas-bearing properties. The brittle mineral content is positively correlated with the gas content (Fig. 10e), while the clay mineral content is positively correlated with the gas content (Fig. 10f). There are primary intergranular pores, dissolved pores, and microcracks related to brittle minerals, which are conducive to the storage of shale gas, especially free gas^22^. In contrast, plastic clay minerals can be compressed and deformed under the action of stress and can increasingly fill pores, which is not conducive to pore preservation^57^. Therefore, a mineral composition with a high brittle mineral content and low clay content improves the gas-bearing property of shale. Finally, based on the results of principal component analysis and geological analysis, the main controlling factors of shale gas-bearing properties are pore volume, SSA, and clay mineral content. The secondary controlling factors are TOC content, brittle mineral content, temperature and pressure. Since temperature and pressure are external factors, this study will not focus on them.

**Figure 11:**
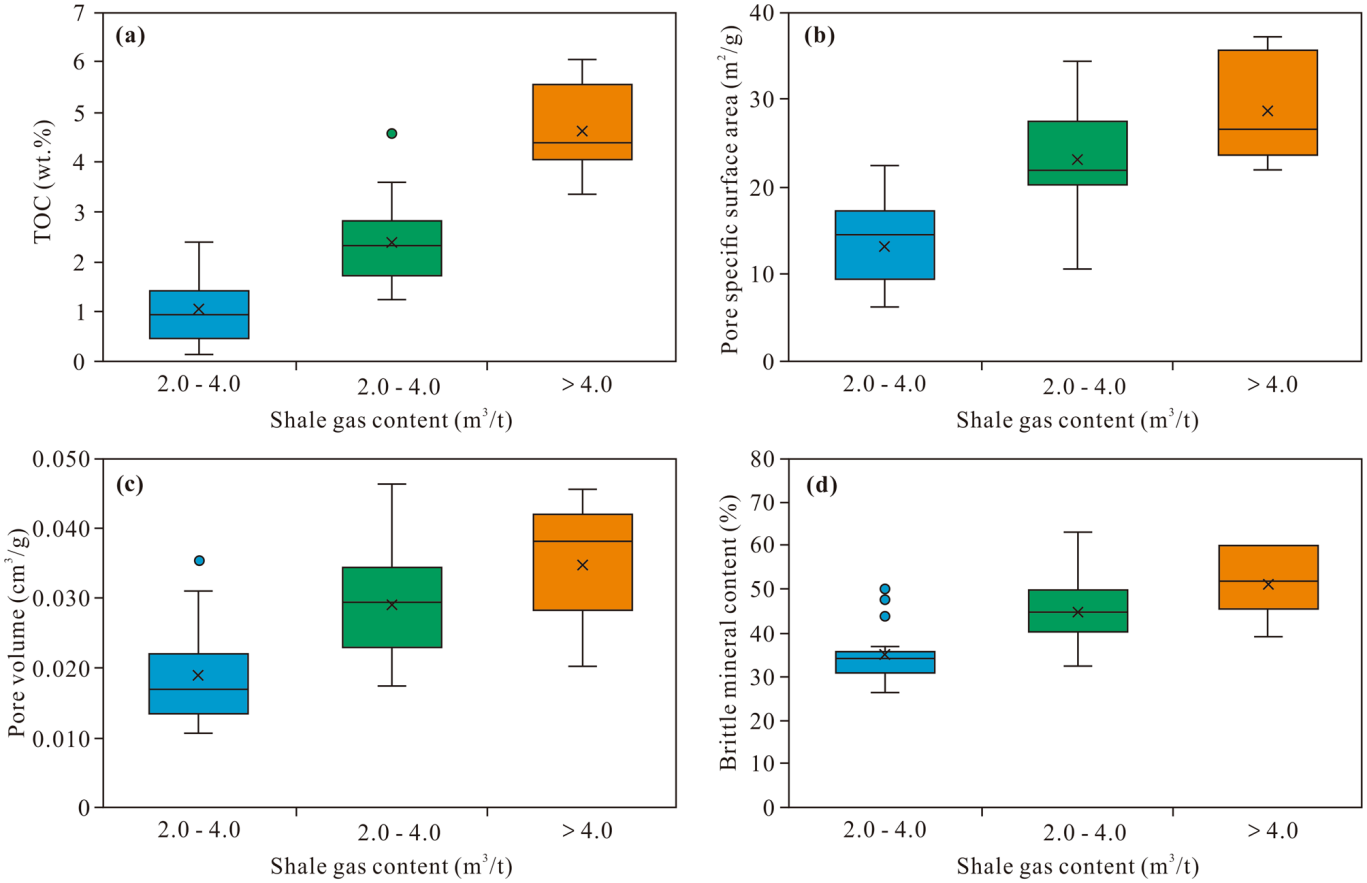
Characteristics of the TOC content (**a**), SSA (**b**), pore volume (**c**), and brittle mineral content (**d**) of the different gas-bearing shale samples.

Statistical analysis demonstrates that the Longmaxi shale samples with good gas-bearing properties exhibit TOC values of 1.94–6.04 wt% (avg. 4.17 wt%), SSA values of 21.846–37.011 m^2^/g (avg. 29.032 m^2^/g), pore volume values of 0.020–0.046 m^3^/g (avg. 0.035 m^3^/g), and brittle mineral contents of 49–75% (avg. 65%). The Longmaxi shale samples with medium gas-bearing properties exhibit TOC values of 1.23–4.57 wt% (avg. 2.33 wt%), SSA values of 10.659–34.301 m^2^/g (avg. 23.002 m^2^/g), pore volume values of 0.018–0.046 m^3^/g (avg. 0.029 m^3^/g), and brittle mineral contents of 41–79% (avg. 56%). The Longmaxi shale samples with poor gas-bearing properties exhibit TOC values of 0.06–2.39 wt% (avg. 0.97 wt%), SSA values of 6.304–22.429 m^2^/g (avg. 12.967 m^2^/g), pore volume values of 0.011–0.036 m^3^/g (avg. 0.019 m^3^/g), and brittle mineral contents of 33–63% (avg. 44%). It is thus clear that the Longmaxi shale samples with good gas-bearing properties exhibit a high TOC content, large pore space, and high brittle mineral content. In contrast, shale with poor gas-bearing properties exhibits the opposite trend (Fig. 11).

### Guidance for shale gas exploration

**Figure 12:**
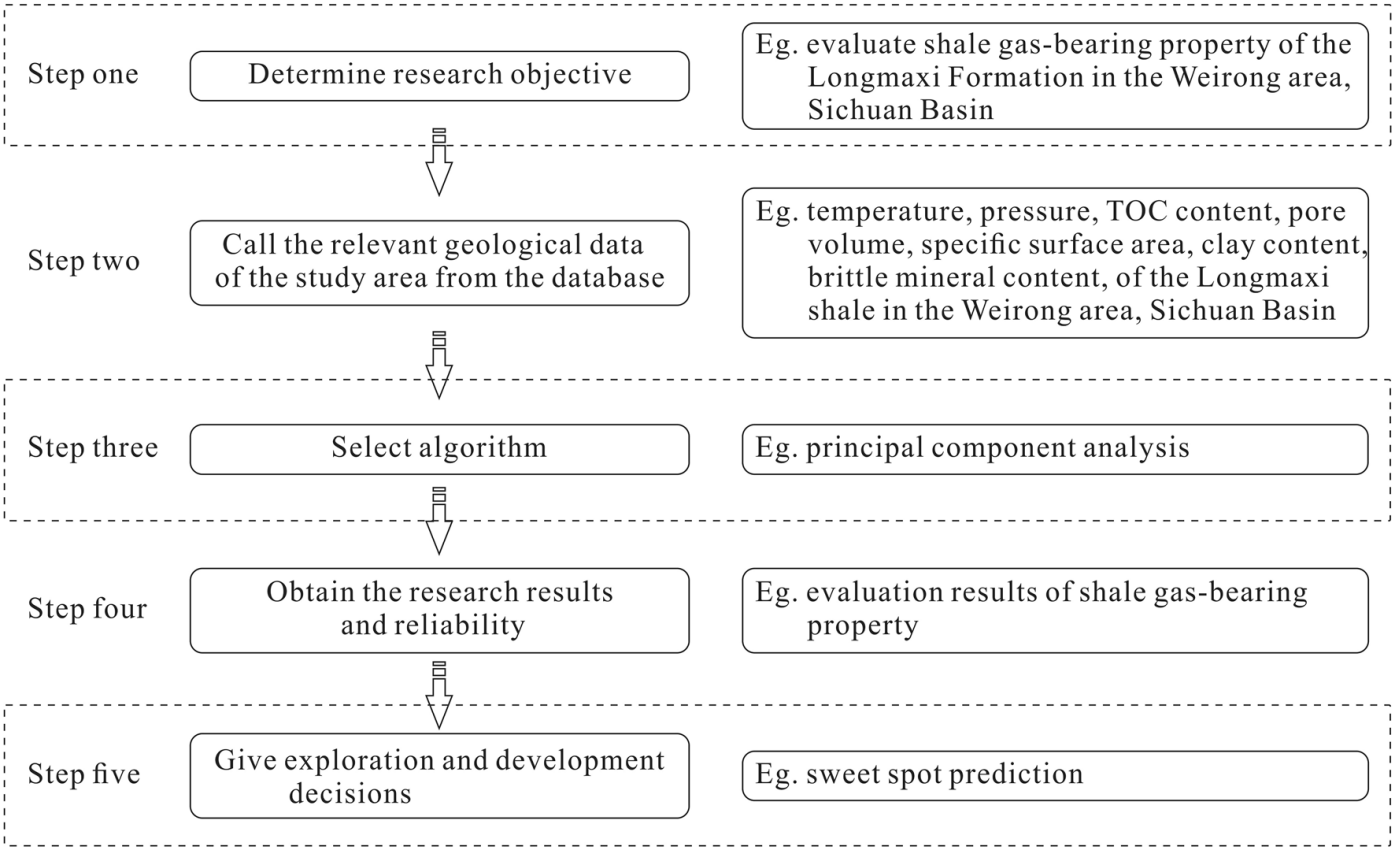
Flow diagram of digital and intelligent shale gas exploration, choosing shale gas-bearing evaluation as an example.

In the era of big data, it is inevitable that digital and intelligent oilfields are constructed, which is very beneficial to the optimization of oilfield exploration and development decisions and improvement in economic benefits^58^. The Longmaxi Formation is an important shale gas-producing layer in China. After decades of research, abundant basic geological data of the Longmaxi shale are available, which provides essential support for the establishment of a vast geological database. A geological database comprises an indispensable part of digital and intelligent oilfields. How can we effectively use a comprehensive geological database to guide digital and intelligent exploration of shale gas? The answer lies in the realization of data analysis intelligence, i.e., the compilation of algorithms. Principal component analysis is a basic, simple, and efficient algorithm. In this paper, effective evaluation of shale gas-bearing properties was realized using principal component analysis, which can provide guidance for digital and intelligent shale gas exploration (Fig. 12). Specifically, four principal components with geological significance, such as the pore structure, formation condition, pore structure, and organic matter abundance, are extracted through principal component analysis in this study. The shale gas-bearing property is evaluated according to the score of the comprehensive factor comprising these four principal components. Gas-bearing property evaluation is the result of comprehensively considering the above geological factors, which are generally also considered in sweet spot prediction of shale gas^59,60^. Therefore, the gas-bearing property evaluation results obtained by principal component analysis can be used as a comprehensive index for shale gas sweet spot prediction. Combined with other geological indicators in the geological database, such as regional structural characteristics, shale physical properties, and surface conditions^61^, the superposition analysis is carried out to realize the graded evaluation of sweet spots in the study area.

## Conclusions

The principal component analysis method can effectively evaluate the gas-bearing properties of the Longmaxi shale, with an accuracy of approximately 80%. Moreover, combining this method and geological analysis, the main controlling factors, namely, SSA, pore volume, and clay content, of gas-bearing properties can be determined. According to the gas-bearing characteristics, it can be considered that the Longmaxi Formation contains three levels of shale gas reservoirs with good, medium, and poor gas bearing properties. Good gas-bearing shale exhibits a gas content > 4 m^3^/t and a comprehensive score > 0.788, with an average TOC content of 4.17 wt%, average SSA of 29.032 m^2^/g, average pore volume of 0.035 m^3^/g, and average brittle mineral content of 65%. Medium gas-bearing shale exhibits a gas content of 2–4 m^3^/t and a comprehensive score of − 0.209 to  0.788, with average TOC, SSA, pore volume, and brittle mineral contents of 2.33 wt%, 23.002 m^2^/g, 0.029 m^3^/g, and 56%, respectively. Poor gas-bearing shale exhibits a gas content < 2 m^3^/t and a comprehensive score < − 0.209, with average TOC, SSA, pore volume, and brittle mineral contents of 0.97 wt%, 12.967 m^2^/g, 0.019 m^3^/g, and 44%, respectively.

In addition, principal component analysis can be used as an algorithm to realize intelligent analysis. The application of principal component analysis in gas-bearing property evaluation lays a foundation for intelligent shale gas exploration, which is an inevitable trend in the era of big data.

## Supplementary Information

Supplementary Table 1.

## Data Availability

All data generated or analyzed during this study are included in this published article.
