# Supplementary material for: Application of mathematical statistics to shale gas-bearing property evaluation and main controlling factor analysis
Source: Sci Rep. 2022 Jun 14;12:9859. doi: 10.1038/s41598-022-13863-1 (PMC9197838; doi:10.1038/s41598-022-13863-1)
Supplement: Supplementary file 1 — Supplementary Table 1. [file 41598_2022_13863_MOESM1_ESM.pdf]

Application of mathematical statistics to shale gas-bearing property evaluation and main controlling factor analysis

Min Li[a, b]; Xiongqi Pang[a, b,*]; Liang Xiong[c]; Tao Hu[a, b]; Di Chen[a, b]; Zhen Zhao[a, b]; Shasha Hui[a, b]

[a] State Key Laboratory of Petroleum Resources and Prospecting, China University of Petroleum (Beijing), Beijing 102249, China;

[b] College of Geosciences, China University of Petroleum (Beijing), Beijing 102249, China;

[c] Research Institute of Exploration and Development, SINOPEC Southwest Branch Company, Chengdu 610041, China.

* Corresponding authors:

E-mail address: pangxq@cup.edu.cn (X.Q. Pang); liminupcqd@163.com (M. Li).

Appendix Supplementary data

Supplementary Table 1 Geological database of the Longmaxi Formation, Southern Sichuan Basin

Samples	Depth (m)	Formation condition		TOC (wt.%)	Mineral composition					Pore structure	
		Temperature (K)	Pressure (MPa)		Clay (%)	Quartz (%)	Feldspar (%)	Carbonate (%)	Brittle mineral (%)	Pore volume (cm^3/g)	Specific surface area (m^2/g)
WY11-1	3688.80	415.62	71.56	0.25	56	34	4	2	40	0.012	7.187
WY11-2	3701.10	416.03	71.80	0.44	57	32	4	1	37	0.013	8.712
WY11-3	3707.30	416.23	71.92	0.13	59	33	4	1	38	0.011	6.304
WY11-4	3711.40	416.37	72.00	0.43	57	32	5	2	39	0.012	7.722
WY11-5	3715.90	416.52	72.09	0.82	53	35	4	1	40	0.014	10.155
WY11-6	3719.18	416.63	72.15	0.92	50	36	4	5	45	0.016	11.342
WY11-7	3720.50	416.67	72.18	0.81	48	35	4	5	44	0.016	9.897
WY11-8	3724.72	416.81	72.26	1.25	52	36	4	3	43	0.016	12.661
WY11-9	3729.36	416.96	72.35	0.83	49	36	5	3	44	0.013	9.088
WY11-10	3732.01	417.05	72.40	0.92	55	34	4	2	40	0.015	10.887
WY11-11	3735.40	417.17	72.47	1.24	51	37	3	4	44	0.018	13.215
WY11-12	3737.65	417.24	72.51	1.33	50	34	3	8	45	0.018	14.396
WY11-13	3742.90	417.41	72.61	2.16	33	30	2	30	62	0.021	17.810
WY11-14	3745.90	417.51	72.67	2.59	36	36	2	22	60	0.023	21.359
WY11-15	3748.25	417.59	72.72	2.76	26	26	3	40	69	0.021	19.530
WY11-16	3752.65	417.74	72.80	2.39	32	35	4	24	63	0.023	19.214

WY11-17	3754.34	417.79	72.83	2.01	35	34	3	23	60	0.019	16.986
WY11-18	3755.60	417.84	72.86	2.31	40	33	3	19	55	0.023	19.745
WY11-19	3756.73	417.87	72.88	2.72	39	32	6	20	58	0.021	20.365
WY11-20	3757.70	417.91	72.90	1.46	44	32	3	18	53	0.022	18.466
WY11-21	3758.92	417.95	72.92	1.90	36	27	2	26	55	0.016	14.551
WY11-22	3759.80	417.98	72.94	1.89	37	29	2	25	56	0.018	15.489
WY11-23	3761.46	418.03	72.97	3.07	40	44	4	4	52	0.024	20.526
WY11-24	3763.52	418.10	73.01	4.34	28	41	1	26	68	0.028	25.987
WY11-25	3764.92	418.15	73.04	4.38	25	18	1	46	65	0.020	23.606
WY11-26	3766.16	418.19	73.06	3.14	28	39	2	26	67	0.025	20.513
WY23-1	3779.08	418.73	73.38	0.08	52	39	4	2	45	0.017	10.842
WY23-2	3782.60	418.90	73.48	0.06	58	34	4	1	39	0.017	9.575
WY23-3	3787.63	419.03	73.56	0.39	61	28	4	1	33	0.022	12.781
WY23-4	3791.53	419.22	73.66	0.37	57	34	3	2	39	0.022	13.460
WY23-5	3797.16	419.35	73.74	1.83	52	35	4	3	42	0.036	22.429
WY23-6	3801.17	419.51	73.84	1.06	50	35	4	4	43	0.028	17.970
WY23-7	3805.94	419.62	73.90	1.49	52	35	4	4	43	0.030	19.925
WY23-8	3809.41	419.69	73.94	1.32	48	40	4	2	46	0.031	18.949
WY23-9	3811.40	419.73	73.97	1.23	55	31	4	6	41	0.029	20.435
WY23-10	3812.78	419.79	74.00	1.37	53	36	4	2	42	0.033	20.687
WY23-11	3814.56	419.84	74.03	1.52	47	36	3	10	49	0.032	21.905
WY23-12	3815.85	419.89	74.06	2.33	51	34	4	5	43	0.036	27.666
WY23-13	3817.55	419.93	74.08	2.57	40	25	4	27	56	0.030	23.572
WY23-14	3818.68	419.99	74.12	2.30	35	24	3	35	62	0.036	27.529
WY23-15	3820.40	420.04	74.15	1.78	32	27	2	35	64	0.046	34.301
WY23-16	3822.10	420.09	74.17	1.65	54	18	3	20	41	0.042	33.735

WY23-17	3823.38	420.17	74.22	2.21	38	27	3	28	58	0.034	27.233
WY23-18	3825.76	420.23	74.26	2.80	36	32	3	24	59	0.031	23.978
WY23-19	3827.69	420.27	74.28	2.33	34	29	2	32	63	0.032	27.519
WY23-20	3828.82	420.39	74.35	4.24	44	34	3	12	49	0.046	37.011
WY23-21	3832.40	420.45	74.38	3.74	39	37	2	18	57	0.039	31.827
WY23-22	3834.20	420.53	74.43	1.94	34	21	1	39	61	0.029	21.846
WY23-23	3836.72	420.57	74.45	3.58	48	31	2	12	45	0.036	31.010
WY23-24	3837.82	420.61	74.48	3.03	42	42	3	7	52	0.034	25.943
WY23-25	3839.17	420.65	74.50	1.70	48	35	6	6	47	0.026	18.879
WY23-26	3840.23	420.67	74.51	2.20	40	35	6	14	55	0.028	21.954
WY23-27	3840.96	420.70	74.53	1.71	41	28	4	23	55	0.027	20.450
WY23-28	3841.75	420.74	74.55	2.31	37	31	6	20	57	0.029	21.910
WY23-29	3842.93	420.77	74.57	2.34	34	44	10	9	63	0.029	23.342
WY23-30	3843.88	420.87	74.63	6.04	19	69	1	5	75	0.042	29.738
WY23-31	3846.92	420.92	74.66	4.57	30	31	1	31	63	0.034	27.930
WY23-32	3848.46	420.93	74.67	5.35	22	57	1	17	75	0.038	35.629
WY23-33	3848.84	421.00	74.71	1.43	18	27	1	51	79	0.018	10.659
WY23-34	3850.84	421.03	74.72	3.34	30	42	2	23	67	0.039	26.610
WY23-35	3851.68	415.62	71.56	2.94	36	34	2	24	60	0.034	25.608
